# Feeling of presence in dementia with Lewy bodies is related to reduced left frontoparietal metabolism

**DOI:** 10.1007/s11682-018-9997-7

**Published:** 2018-12-04

**Authors:** Nicolas Nicastro, Antoine F. Eger, Frederic Assal, Valentina Garibotto

**Affiliations:** 1 Department of Psychiatry, Addenbrooke’s Hospital, University of Cambridge, Hills Road, Cambridge, CB2 0QQ UK; 2 Division of Neurorehabilitation, Department of Clinical Neurosciences, Geneva University Hospitals, Geneva, Switzerland; 3 Division of Neurology, Department of Clinical Neurosciences, Geneva University Hospitals, Geneva, Switzerland; 4 Department of Nuclear Medicine, Geneva University Hospitals, Geneva, Switzerland; 5 NiMTLab, Faculty of Medicine, University of Geneva, Geneva, Switzerland

**Keywords:** Psychosis, Dementia, Brain glucose metabolism, Statistical parametric mapping

## Abstract

Feeling of presence (FOP) refers to the vivid sensation of a person’s presence near oneself and is common in Dementia with Lewy Bodies (DLB). Based on previous observations on epileptic subjects, we hypothesized that DLB subjects with FOP would harbour ^18^F-fluorodeoxyglucose PET hypometabolism in left parietal areas. 25 subjects (mean age 71.9 ± 6.7, disease duration at scan 1.7 ± 1.5 years) were included in the study, of whom nine (36%) experienced FOP. No significant between-group difference was observed regarding dopamine transporters striatal uptake (*p* = 0.64), daily dopaminergic treatment dosage (*p* = 0.88) and visual hallucinations (*p* = 0.83). Statistical parametric mapping showed that subjects with FOP had a significantly reduced glucose metabolism in several left frontoparietal areas (*p* < 0.001), including superior parietal lobule and precuneus. Interregional correlation analysis of these areas showed specific connectivity with right insula and putamen in the FOP subgroup and right orbitofrontal and superior frontal in subjects without FOP. This provides further evidence about the role of a left frontoparietal network and suggest a possible contribution of impaired orbitofrontal reality filtering associated with FOP.

**Electronic supplementary material:**

The online version of this article (10.1007/s11682-018-9997-7) contains supplementary material, which is available to authorized users.

## Introduction

Dementia with Lewy bodies (DLB) is the second most common degenerative dementia after Alzheimer’s disease (AD), accounting for 15% of cases (Vann Jones and O'Brien 2014; Zaccai et al. 2005). It is characterized by a progressive cognitive decline interfering with activities of daily living in association with core clinical features of parkinsonism, visual hallucinations (VH), cognitive fluctuations and REM sleep behavior disorder. The recently revised diagnostic criteria (McKeith et al. 2017) have integrated decreased posterior metabolism on ^18^F-FDG-PET and considered as an indicative biomarker the presence of reduced dopaminergic uptake in the basal ganglia on ^123^I-FP-CIT single photon emission computed tomography (SPECT) imaging in order to increase diagnostic accuracy and provide specific management considerations (Boot 2015; O'Brien et al. 2014; Thomas et al. 2017). Although there is no available specific marker of Lewy body pathology (Bauckneht et al. 2017), dopaminergic SPECT provides excellent sensitivity and specificity (resp. 78 and 90%) to discriminate neuropathology-confirmed cases of DLB (McKeith et al. 2007) from other dementias like AD. Adding semiquantitative assessment to the standard visual analysis allows even greater accuracy (Nicastro et al. 2017).

Feeling of presence (FOP) refers to the vivid sensation that somebody is present near oneself in the absence of any sensory clue. It has been described in various conditions such as schizophrenia (Koehler and Sauer 1984), epilepsy (Arzy et al. 2006; Blanke et al. 2003), Parkinson’s disease (Fenelon et al. 2000), and DLB (Nagahama et al. 2010). FOP is the most frequently encountered psychotic symptom in patients with Parkinson’s disease (34–44%) (Fenelon et al. 2011; Williams et al. 2008). It is usually short-lived (a few seconds), rarely distressing, and although patients usually have a preserved insight about the “fictitious” nature of this feeling, they have a tendency to check for a real presence. FOP usually occurs behind or beside the subject, without any side predominance (Fenelon et al. 2011). Independent predictors include concomitant VH and possibly higher daily levodopa dosage. Besides, Nagahama et al. (2010) have described significant ^99m^Tc HMPAO SPECT hypoperfusion of bilateral angular gyrus, left fourth occipital gyrus and right supramarginal gyrus in DLB patients experiencing cluster symptoms of VH and FOP. Right occipitotemporal hypometabolism has been described in subjects with visual hallucinations (Perneczky et al. 2008).

With the present study, we aimed at evaluating specific FDG-PET hypometabolism in a retrospective cohort of subjects with probable DLB, based on statistical parametric mapping (SPM) analysis. Considering observations on epileptic patients experiencing FOP during electrostimulation or seizure (Arzy et al. 2006; Blanke et al. 2003; Picard 2010; Zijlmans et al. 2009), our hypothesis was that DLB subjects with FOP (FOP+) would have a reduced metabolism in left temporoparietal cortex in comparison to those without FOP (FOP-). In addition, we aimed at determining whether FOP+ have a higher levodopa equivalent daily dose (LEDD) and a reduced presynaptic dopamine SPECT uptake in comparison to FOP-.

## Method

### Participants

The present study has been conducted in compliance with the declaration of Helsinki and our local Ethics Committee has approved its protocol (NAC 12-026R). We collected clinical data (gender, age and disease duration at scan, clinical diagnosis) from all subjects for which both ^123^I-FP-CIT SPECT and ^18^F-FDG PET were available for analysis, provided they were performed at a maximum of 2 months apart. We considered all subjects who underwent scans from October 2003 to October 2016 within Geneva University Hospitals with the same acquisition and image processing protocols. We then included all patients harbouring a clinical diagnosis of probable DLB according to the most recent consensus criteria of McKeith et al. (2017). Although FDG PET is not necessary for the diagnosis of DLB, we have been able to collect this imaging data for our cohort either because the subjects were first evaluated for suspected AD (visuospatial impairment with no or mild parkinsonism), or because ^123^I-FP-CIT SPECT only showed moderate presynaptic DAT impairment, thus requiring another imaging evaluation to confirm a posterior hypometabolism evoking DLB.

Based on the information in the medical file, we searched for FOP description (presence, side predominance), as well as clinical features possibly associated with FOP, such as severity of parkinsonism based on Movement Disorders Society Unified Parkinson’s Disease Rating Scale (MDS-UPDRS III) (Goetz et al. 2008), cognitive decline (Mini-Mental State Evaluation, MMSE, (Folstein et al. 1975)) and levodopa equivalent daily dose (LEDD, (Tomlinson et al. 2010)).

### PET and SPECT imaging acquisition and reconstruction

#### FDG-PET

Subjects received 250 MBq of ^18^F-FDG in slow IV injection under standardized conditions (supine position, low ambient noise, dimly- lit room, eyes open). Usual medication schemes were continued before and on the day of scan. PET/CT data acquisition was performed on a Biograph tomograph (Siemens Healthcare, Erlangen, Germany) using manufacturer’s recommendations and according to the European Association of Nuclear Medicine Neuroimaging Committee (Varrone et al. 2009). PET acquisition started approx. 30 min after the ligand injection. CT study was used for attenuation and scatter correction, then followed by PET emission study (20 min). We used an ordered-subset-expectation-maximisation (OSEM) algorithm for image reconstruction.

#### FP-CIT SPECT

^123^I-FP-CIT SPECT was performed according to the manufacturer’s instructions. Patients received 185 MBq of ^123^I-FP-CIT (ioflupane, DaTSCAN®, GE Healthcare) in slow IV injection and Lugol solution (or sodium perchlorate) for thyroid blockade. SPECT data acquisition started 4 h after ioflupane injection. Acquisition was performed on the same triple-head gamma camera (GCA-9300A/UI Toshiba Medical Systems AG, Oetwil am See, Switzerland) equipped with fan beam low-energy high-resolution collimators. Details of the acquisition and reconstruction are available in (Nicastro et al. 2016b).

##### SPM for PET

FDG-PET brain images were pre-processed using Statistical Parametric Mapping (SPM12, Wellcome Trust Centre for Neuroimaging, London, UK, http://www.fil.ion.ucl.ac.uk/spm/software/SPM12/), running in Matlab R2017a Version 9.2.0 (MathWorks Inc., Sherborn, MA). We performed approximate manual image re-orientation and positioning to a T1 MRI template available in SPM toolbox. Images were spatially normalized and written in the default SPM12 bounding box with an isotropic voxel size of 2 × 2 × 2 mm. Visual inspection of normalized images allowed to ensure registration quality and convergence of the procedure. Image smoothing was performed with an isotropic 3D Gaussian kernel of 8 mm full width at half-minimum (FWHM). We performed a two-sample t-test (subjects with and without FOP) with Analysis of Covariance (ANCOVA) design, corrected for age and gender. Intensity normalization was performed using the individual pontine metabolism as it has been shown not to be affected in subjects with dementia (Borghammer et al. 2008; Minoshima et al. 1995). T-maps contrasts were obtained by comparing DLB subjects with (−1) and without FOP (+1), with an uncorrected threshold at 0.001. To report corresponding brain areas, we used coordinates from Montreal Neurological Institute (MNI) (Mazziotta et al. 1995) and Wake Forest University (WFU) Pickatlas Software Version 3.0.5 toolbox running on SPM12 (Maldjian et al. 2003). In addition, we performed for both subgroups interregional correlation analysis (IRCA) (Lee et al. 2008) of extracted mean regional volumes of interest counts (normalized with respect to pontine mean counts) as covariate to find regions presenting significant voxel-wise positive and negative correlations with the ones showing significant hypometabolism in FOP+ group (uncorrected *p* < 0.001, extent threshold k = 20 voxels). This method has been used in subjects with AD (Morbelli et al. 2012). As left temporoparietal junction (TPJ) has been shown to induce FOP in epileptic subjects, we also assessed IRCA of TPJ by creating a spherical 10 mm-mask with MNI coordinates as found in the publication of Sowden et al. (Sowden and Catmur 2015). For both subgroups, IRCA of adjacent regions to TPJ was also studied (left angular, supramarginal, and superior temporal gyri).

##### Reference limits for SPECT

We collected standard 0-to-3 (Benamer et al. 2000) visual staging in the initial nuclear medicine report available in the patients’ medical file. Site-specific semiquantitative reference values have been established in a previous publication and formulae to determine striatal, caudate nucleus and putaminal uptake limits (Nicastro et al. 2016a) have been applied to assess specific uptake of included subjects, expressed in %.

### Statistical analysis

We used Stata Version 14.2 software (College Station, TX) for statistical analysis. Continuous variables were tested for normality with the Shapiro-Wilk test. We used t-test for continuous variables with a normal distribution, Wilcoxon Rank Sum (Mann-Whitney U Test) for non-parametric variables and Chi-Square test for discrete variables accordingly. Statistical significance was considered if *p* value <0.05. We reported values as mean ± standard deviation (SD) (range).

## Results

### Patient demographics and clinical caracteristics

We identified 25 subjects fulfilling McKeith diagnostic criteria of probable DLB for which both ^18^F-FDG PET and ^123^I-FP-CIT SPECT were performed according to the aforementioned criteria. Baseline characteristics of included subjects are available in Table 1. For the whole group, age was 71.9 ± 6.7 years (range 58–86), with 32.0% female (8/25) and disease duration 1.7 ± 1.5 years (0.25–7). Among these, we identified nine FOP+ and 16 FOP-. There was no statistical difference between both subgroups regarding age, disease duration, and male/female ratio. In addition, MMSE score, presence of visual hallucinations and ^123^I-FP-CIT SPECT visual and semiquantitative assessments did not differ between FOP+ and FOP- subjects. Similarly, there was no statistical difference between both subgroups regarding LEDD and MDS-UPDRS III scores.

**Table 1:** Baseline characteristics

	All DLB	FOP	No FOP	Pval
#	25	9	16	
Age (years)	71.9 ± 6.7 (58–86)	71.2 ± 6.9 (58–78)	72.3 ± 6.8 (61–86)	0.70^#^
Female Gender %	32.0% (8/25)	22.2% (2/9)	37.5% (6/16)	0.43^&^
Disease duration (years)	1.7 ± 1.5 (0.25–7)	2.1 ± 2.2 (0.25–7)	1.5 ± 1.0 (0.25–3)	0.62*
MMSE	21.6 ± 4.8 (11–26)	22.8 ± 5.6 (13–26)	21.1 ± 4.5 (11–26)	0.19*
MDS-UPDRS III	13.1 ± 14.0 (0–52)	11.7 ± 14.1 (0–44)	13.9 ± 14.4 (0–52)	0.68*
LEDD (mg)	112 ± 233 (0–800)	109.8 ± 266.2 (0–800)	113.3 ± 221.6 (0–750)	0.88*
Visual Hallucinations	64% (16/25)	66.7% (6/9)	62.5% (10/16)	0.83^&^
Dopamine SPECT visual stage	1.9 ± 0.9 (0–3)	2.0 ± 0.7 (0–3)	1.9 ± 1.1 (0–3)	0.82^&^
Mean striatal dopamine SPECT uptake	1.79 ± 0.64 (0.77–2.91)	1.71 ± 0.55 (0.88–2.62)	1.84 ± 0.70 (0.77–2.91)	0.64^#^
% mean striatal dopamine SPECT uptake (according to reference limits)	75.1 ± 25.7 (33.5–117)	71.2 ± 21.4 (37.5–97.8)	77.4 ± 28.2 (33.5–117)	0.57^#^

Baseline characteristics of DLB subjects included in the study. Values are mean ± SD (range). Statistical tests (FOP+ vs FOP- subgroups): * Wilcoxon Rank Sum (Mann-Whitney U), # Student T, & Chi-Squared

### FDG PET analyses

#### Group comparison

Regarding the FOP subgroup, 6/9 (66.7%) subjects felt the presence beside them and 3/9 (33.3%) behind them. When FOP was felt beside the patient, it was perceived on right (2/6), left (1/6) or both sides (3/6).

**Fig. 1:**
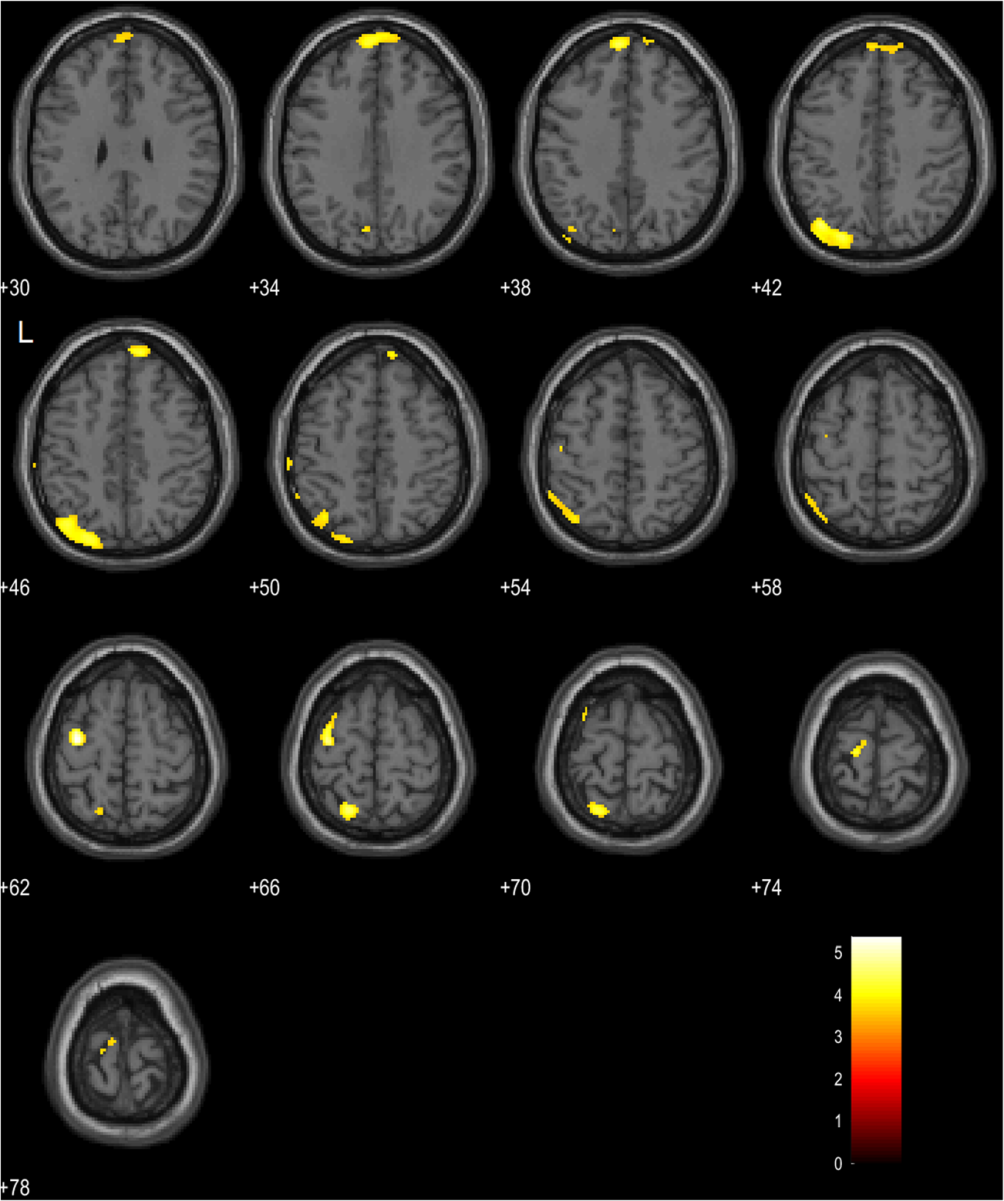
Slice overlay of the major hypometabolic clusters in the FOP+ group (uncorrected *p* < 0.001). The SPM(Z) result image has been overlaid on a template T1 MRI. Numbers indicate the Z coordinates according to MNI. L = left. Scale shows T-Score

FOP+ showed significantly more pronounced hypometabolism compared with FOP- in left parietal (superior parietal lobule and precuneus) as well as frontal areas (middle, superior and precentral gyri) (uncorrected *p* < 0.001) (Table 2**;** Fig. 1). No significant clusters survived false discovery rate (FDR) or family-wise error (FWE) *p* < 0.05 correction.

**Table 2:** Hypometabolic clusters associated with FOP

Cluster	Region	Voxels	x y z	Z-score
1	Middle frontal gyrus, left	79	−34 -10 62	4.14
2	Superior parietal lobule, left	30	−18 -60 66	3.75
3	Precuneus, left	112	−40 -72 42	3.71
4	Superior frontal gyrus, left	98	−4 58 38	3.68
5	Precentral gyrus, left	34	−16 -20 74	3.54

Brain regions showing significant hypometabolism in relation to FOP. X Y Z = stereotactic coordinates in MNI space; Z score = SPM(Z) score

#### Connectivity analysis

**Fig. 2:**
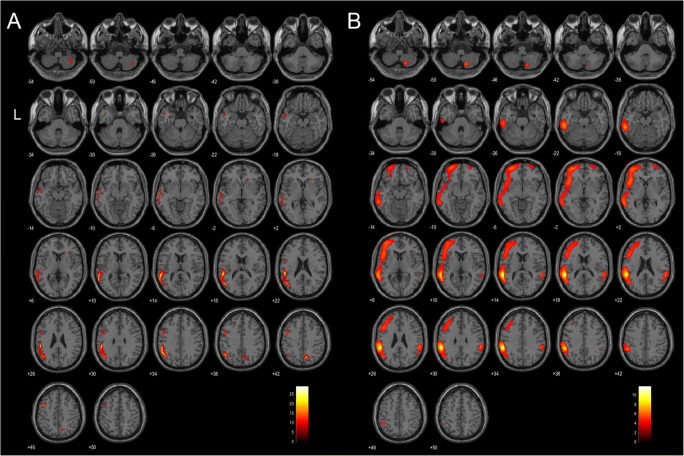
Slice overlay for FOP+ (*part A*) and FOP- (*part B*) subjects showing interregional correlation analysis (IRCA) with left supramarginal gyrus (positive correlation, uncorrected p < 0.001, extent threshold >20 voxels). FOP- subjects show a more extended connectivity, including bilateral superior and orbitofrontal gyri, as well as contralateral supramarginal gyrus. Numbers indicate Z coordinates according to MNI. Scale represents T-Score. L = left

In both subgroups, IRCA of the seed regions included large clusters with a significant positive correlation, encompassing left temporoparietal junction, left superior and mid frontal gyri, as well as the right 8th cerebellar hemisphere. Specific IRCA was found for the FOP+ group in right putamen, insula and lingual gyrus (Table 3). On the other hand, FOP- subjects specifically showed right orbitofrontal and superior frontal connectivity (Fig. 2).

**Table 3:** Interregional correlation analysis of hypometabolic clusters and putative regions implicated in FOP

	FOP+ subjects (*n* = 9)	FOP- subjects (*n* = 16)
Left Middle Frontal	L middle Frontal (*autocorrelation*) L superior Frontal R Superior Temporal **R Insula** L angular **R lingual** R Cerebellum (8th hemisphere)	L middle Frontal (*autocorr.*) L middle Temporal R Supramarginal and middle Temporal R Cerebellum (8th hem.)
L Superior Frontal	L superior Frontal (*autocorr.*) L Superior Temporal **R and L Insula** R and L Precuneus **R lingual**	L and R Superior and Middle Frontal (*autocorr.*) L and R Inferior Temporal L and R angular and supramarginal R Cerebellum (8th hem.)
L Precentral	L middle Frontal L Precentral (*autocorr.*) L anterior Cingulate L Rolandic Operculum R and L Superior Parietal **R Putamen**	L and R Precentral (*auto*) L and R middle Frontal **R superior Frontal** L Rectus L inferior Temporal L Superior Parietal
L Precuneus	L Superior Frontal R Precentral L middle Temporal **R Insula** L and R Precuneus (*autocorr.*) L Putamen	**R superior Frontal** L middle Frontal L and R Precuneus and superior Parietal (*autocorr.*)
L Superior Parietal	L Superior Frontal R middle Temporal pole R Superior Parietal L inferior and superior Parietal (*autocorr.*)	**R superior Frontal** L superior Parietal and Precuneus (*autocorr.*)
L angular	L angular, supramarginal and middle Temporal (*autocorr.*) R Precuneus R Supramarginal **R Putamen**	Middle Temporal, angular and inferior Parietal (*autocorr.*)
L supramarginal	L Precentral **R Insula** L angular, supramarginal, superior and middle Temporal (*autocorr.*) R Precuneus R Cerebellum (8th hem.)	L Inferior and superior Frontal **R middle and superior Orbitofrontal** R middle Temporal R Cerebellum (8th hem.)
L Superior Temporal	L Superior and middle Temporal (*autocorr.*) **R Insula** R Cerebellum (8th hem.)	L and R middle and superior Temporal (*autocorr.*) R Cerebellum (8th hem.)
L Temporoparietal junction	L Superior Frontal L Precentral **R Insula** L angular, supramarginal and Superior Temporal (*autocorr.*) R Precuneus R Cerebellum (8th hem.)	L and R middle Frontal L middle Temporal, angular and supramarginal (*autocorr.*) R supramarginal

Interregional Correlation Analysis (IRCA) of the five hypometabolic FDG PET clusters in subgroup with FOP, as well as putative areas implicated in FOP (left temporoparietal junction and adjacent regions) used as seeds. Positive correlation, uncorrected p < 0.001, extent threshold >20 voxels. L = left, R = right

The IRCA did not identify significant negative correlations with these regions.

## Discussion

FOP is frequently encountered in subjects with DLB, as already shown by other authors (Nagahama et al. 2010). In the present study, we found that 36% (9/25) of retrospectively evaluated probable DLB patients experienced such symptoms. We observed that FOP was perceived behind (33.3%) of beside (66.7%) the subject, without any side predominance in the latter case. Interestingly, subgroups with and without FOP have similar age, disease duration, as well as cognitive (MMSE) and motor impairment (MDS-UPDRS III). At variance with another study including PD subjects (Fenelon et al. 2011), VH was not a predictor of FOP in our DLB population. One hypothesis could be that in the case of PD subjects, both VH and FOP should be considered as symptoms of severe and widespread cortical damage (in the study of Fenelon, disease duration of PD patients with FOP was 11 years). In our case, VH are much more prevalent (64% of the whole DLB group), as it is a core clinical feature of DLB. Besides, we observed similar LEDD and UPDRS score in FOP+ and FOP-. At variance with the study of Fenelon, our DLB subjects had a much lower L-Dopa daily intake (mean 112 vs 959 mg/day), so this could be explained either by a smaller effect size or by different mechanisms eliciting FOP in DLB and PD patients.

SPM analysis of FDG-PET showed significantly reduced metabolism in left frontal and parietal areas in the FOP+ subgroup, when compared to FOP- DLB subjects. More specifically, FOP was associated with a more pronounced hypometabolism in precuneus, superior parietal lobule, as well as superior, middle frontal and precentral gyri, all in the left hemisphere (uncorrected *p* < 0.001). fMRI and PET studies suggest a role in visuospatial imagery regarding precuneus (Culham et al. 1998; Malouin et al. 2003). The superior parietal lobule is involved in spatial orientation (Galletti and Fattori 2017) and has been implicated in distinction of body parts in self and non-self (Felician and Romaiguere 2008).

IRCA in the FOP+ group showed that these five clusters were functionally connected to various regions including right putamen, insula and lingual gyrus, whereas FOP- subjects showed a different connectivity including right superior frontal gyrus.

To the best of our knowledge, the neural correlate of FOP in DLB subjects was only studied by Nagahama et al. (2010) using ^99m^Tc HMPAO, with significant hypoperfusion in bilateral angular gyri, left fourth occipital and right supramarginal gyrus. However, it must be emphasized that analyses were performed for a symptom cluster associating VH and FOP, the former accounting (as suggested by the authors) for the major effect as 92% of the group had VH, whereas only 39% presented FOP. There is substantial evidence that VH in patients with PD and DLB are correlated with posterior hypometabolism on FDG-PET, especially in bilateral precuneus and lingual gyrus (Boecker et al. 2007; Gasca-Salas et al. 2016; Perneczky et al. 2008). In addition, occipital and fusiform gyrus SPECT hypoperfusion has been observed (Heitz et al. 2015; Oishi et al. 2005). However, it is unclear whether FOP phenomenon shares similar neural basis with VH.

Cortical electrostimulation in subjects suffering from epilepsy can be enlightening to understand the specific regions implicated in the pathogenesis of FOP. In fact, electrocortical stimulation of the left parietotemporal (Arzy et al. 2006) and parietotemporooccipital junction (Zijlmans et al. 2009) has been described to elicit FOP similar to those experienced by PD and DLB subjects, i.e. the intense and short-lived sensation of a presence next to the patient. Notably, our findings are very close, as the main cluster of hypometabolism in DLB with FOP is located in the left parietal lobe (superior parietal lobule and precuneus), which is near the parietotemporal junction. In addition, IRCA of left TPJ and adjacent areas (left supramarginal, angular and superior temporal gyri) in our FOP+ subgroup shows a connectivity pattern similar to that of hypometabolic regions in FOP+, i.e. a network encompassing left superior and mid Frontal gyrus and right cerebellum. Besides, additional connectivity was observed for FOP- subjects in right superior and orbitofrontal gyri, which are implicated in reality filtering (Schnider 2013). This finding can be considered as a possible explanation for the occurrence of FOP in selected DLB subjects.

However, further speculations cannot be inferred from our study, as it is based on a functional PET analysis aimed at evaluating the baseline metabolism of DLB subjects with or without FOP and not to assess the critical region which is stimulated during the actual experience of FOP.

FOP+ and FOP- subjects show similar striatal uptake on dopamine SPECT with both visual staging and semiquantitative assessment. In our opinion, this finding is not surprising, as FOP presumably represents a symptom of cortical alteration that has been described in patients with epilepsy and schizophrenia, the mechanisms of which are not related to an impairment of dopaminergic pathways.

Several limitations must be addressed regarding the present work. First, as it is the case for the majority of studies on this topic, diagnosis of DLB was not based on the gold standard neuropathological confirmation, but on clinical and PET/SPECT evaluation. We nonetheless used the most recent diagnostic criteria of probable DLB in association with a decreased presynaptic dopaminergic uptake on ^123^I-FP-CIT SPECT and a typical posterior ^18^F-FDG PET hypometabolism. Second, sample size is quite small but we were still able to demonstrate a significantly more pronounced hypometabolism in various clusters for DLB subjects with FOP. With regard to FDG-PET cluster determination, the exploratory nature of the study and the relatively reduced sample allowed us to use a liberal uncorrected *p* < 0.001 threshold and we were not able to replicate these findings with a more stringent FDR or FWE *p* < 0.05 correction. Finally, we cannot exclude that due to the retrospective design of our study, we may have missed additional subjects having experienced FOP but whose information was not notified in the medical file.

DLB subjects experiencing FOP have a reduced FDG-PET metabolism in left frontoparietal areas and a different connectivity map that FOP- subjects. The former present a network including the right putamen and insula, whereas the latter show more extended connections with contralateral homologous areas, as well as with right superior and orbitofrontal areas, known to be associated with reality filtering. On the other hand, presynaptic dopamine SPECT imaging striatal uptake impairment as well as dopaminergic treatment do not seem to be correlated with FOP in our DLB group. These findings provide major insights into the understanding of psychotic symptoms in subjects with dementia and their functional correlation with metabolic imaging, pinpointing to a disrupted left frontoparietal network underpinning FOP.

## Electronic supplementary material

ESM 1 (DOCX 11 kb)
