# Supplementary material for: Feeling of presence in dementia with Lewy bodies is related to reduced left frontoparietal metabolism
Source: Brain Imaging Behav. 2018 Dec 4;14(4):1199–207. doi: 10.1007/s11682-018-9997-7 (PMC7381475; doi:10.1007/s11682-018-9997-7)
Supplement: Supplementary file 1 — (DOCX 11 kb) [file 11682_2018_9997_MOESM1_ESM.docx]

Feeling of presence is frequently encountered in dementia with Lewy bodies

It is not correlated with dopamine treatment or dopamine SPECT imaging

We observed reduced FDG PET metabolism in left frontoparietal areas

Subjects without feeling of presence have a preserved orbitofrontal network
